# Dissertations on Points of Practical Medicine and Surgery

**Published:** 1834-07-01

**Authors:** 


*Abhandlungen aus dem Gebiete der Practischen Medicin und Chirurgie.* Von Dr. Adolph. Leop. Richter, &c.—Berlin, 1832.

*Dissertations on Points of Practical Medicine and Surgery.* By Dr. A. L. Richter. 8vo. pp. 261.

This is an extremely pleasant and instructive volume, consisting of several detached medical and surgical essays, exhibiting a view of what has been previously written on the subjects of which they treat, and interspersed with interesting cases and remarks, which communicate to the whole an air of originality. Papers of this kind, combining literary history with the statement of new facts, are particularly entertaining and useful, and require extensive information, together with considerable talent and judgment, in their author. The work before us evinces all these: it is not, however, altogether free from a fault which pervades the entire medical literature of Germany—that of too great diffuseness, and a disposition to dwell on trifling circumstances. We cannot here refrain from expressing our regret at this unfortunate peculiarity in the German writers; nor is it possible to suppress a feeling of chagrin, when, as frequently happens, after the toilsome enucleation of an author's meaning,—cum sudor ad imos manaret talos, we find that we have waded through a dense volume to come at something which might have been amply set forth in half a dozen pages.

We can assure our Teutonic brethren, that, if they would give themselves only about a twentieth part of the trouble they do, theirs would be the finest medical literature on earth. Their information is immense, their correctness exemplary: all they lack is condensation, which, however, is a most essential requisite for scientific excellence.

We by no means wish to insinuate that these remarks are particularly applicable to Dr. Richter's book, which, if it be not exempt from the charge of unnecessary diffuseness, is, on the whole, less obnoxious to it than most others in the same language: some of the cases, however, are narrated at so tedious a length, that we have been unable to present them to our readers, though desirous of doing so, from their really interesting character.

The subjects treated of in this volume are—

1. The use of gypsum in the treatment of fractures of the leg.
2. The preternatural formation of bone in muscles.
3. The curative process of nature in introsusception.

4. Chronic rheumatic inflammation of the synovial membrane of the elbow-joint.

5. The interstitial absorption of bones.

6. Four interesting cases of disease.

7. The production of modified and spurious small-pox, and of cow-pox, from the contagion of genuine small-pox.

8. Gangrenous erosion of the face in children.

In the first article our author alludes to several recent contrivances for the treatment of fractured legs; as that of Förster, who, after the bones are replaced, lays the leg, with the foot extended, in a box of sufficient length, which is half filled with sand moistened with water. So much sand is then poured on each side of the leg as to surround it to the edges of the shin, which is left free. In the lower end of this box are two little doors, to admit of the extension of the foot. Instead of the footboard Kluge has substituted a valve, perforated with holes for the transmission of the extending cords. The sand, kept constantly moist, forms an equal and comfortable support for the limb without exerting undue pressure; it affords a ready access to the injured part, and has the advantage of being always procured without difficulty or expense. Dr. Richter states, that, although he has seen this plan successfully adopted in many cases of simple and complicated fractures of the leg in the Berlin Hospital, he does not regard it in general as the best mode of treatment: he considers it chiefly applicable to the early or inflammatory period of those cases where there is injury of the soft parts; he also thinks it a desirable means when the accident occurs in the country, and *no other can be obtained.* The latter is always a good reason for taking what one can get, and would be our only motive for using the sand, which appears to us a very slovenly and inefficient proceeding.

The contrivance of Larrey is much neater. Instead of splints, longitudinal folds of linen, soaked in ointment of styrax, are applied over the fractured part; the whole limb is then carefully surrounded with lint and compresses dipped in a glutinous fluid, prepared with wine, or camphorated vinegar, and white of egg, and the whole is retained by an eighteen-tailed bandage. (*Journal Complémentaire du Dictionnaire des Sciences Médicales,* tom. xx.)

To these we may add, a method recommended by the late Mr. Allen, of Edinburgh, who, in the treatment of these and other fractures, made use of pasteboard splints, moistened in warm water, so as to adapt them to the shape of the part, and then soaked in glue, by the hardening of which a light but firm case is formed for the fractured limb. (*System of Pathological and Operative Surgery*, vol. ii. p. 67.)

The earliest account of the use of gypsum which Dr. Richter has met with, is in an extract from a letter of Mr. Eaton, formerly English consul at Bassorah, to Dr. Guthrie, of Petersburgh, contained in the ninth volume of the Medical Commentaries. The practice is here mentioned as being in use among the Arabian surgeons. L. F. v. Froriep also, in his Introduction to Roux's Parallel between English and French Surgery, states the same practice to be prevalent among the Moors on the northern coast of Africa.

"So far as I know," continues Dr. Richter, "a trial of this method of treatment in fractures of the leg has only been made in the hospital at Berlin in the year 1828-9, by order of the director, Professor Kluge, and under the inspection of the house physician, Dr. Keyl. This practice, of whose utility I have also had an opportunity of convincing myself, has been made the subject of an inaugural dissertation by Dr. F. W. Ferd. Rauch, entitled 'De gypso liquefacto ad fracturas ossium cruris curandas adhibendo:' Berolini, 1829. The following detail is given of the mode of proceeding. A wooden box is used, of such a length that the leg and condyles of the femur may be conveniently laid in it. This box is half filled with sand; on the sand is laid a piece of pasteboard six inches broad, and on each side of this a similar piece is stuck into the sand, so that the space thus enclosed indicates the form of the mass of gypsum about to be poured in. These pieces of pasteboard, as well as the calf of the leg, are anointed with oil, or lard, to prevent the adhesion of the gypsum. The leg is then placed, with the foot extended, in the space enclosed by the pieces of pasteboard, and the gypsum is poured quickly and without interruption around the leg till the larger half thereof is surrounded by it. (The gypsum is to be prepared for this purpose by gradually pouring a peck of gypsum into about eight quarts of water, carefully stirring the mixture.)

"In half an hour the gypsum is hardened, and the box as well as the pasteboard may be removed. A more convenient adjustment is afterwards made, by the adaptation of a box, twenty-two inches long by seven broad, whose sides can be let down and removed from the bottom. The hinder wall has also an aperture for the reception of the ham. This box may be used for either leg, and forms a receptacle in which the extended limb may have the gypsum poured around it. After the gypsum is hardened, the sides of the box may be let down, and removed along with the bottom."

The gypsum may, according to the circumstances of the case, be applied in an unbroken mass, as above described, or in detached portions, which admit of being separately removed. Each portion of the gypsum may be made to assume any desired shape, by using a piece of pasteboard, of the proper form, as a mould.

When the fracture is complicated with a wound, an aperture of sufficient size must, of course, be made in the covering of gypsum.

The gypsum is best applied in a continuous mass in cases of simple fracture, where we have little to apprehend from inflammation, or other local consequences. The use of separate portions is more applicable to complicated fractures.

Some more minute directions are given for the preparation and application of the gypsum, for which we must refer to the original memoir.

Dr. Richter details the case of a Captain K—, which he treated with success on the above-mentioned plan. The fracture was situated three inches above the ankle-joint, the bone was splintered, and the injury attended with much contusion, swelling, and extravasation of blood. After the inflammatory symptoms were subdued by appropriate means, the gypsum was applied in separate portions, and at the expiration of sixteen weeks the patient was able to return to his duty.

All ingenious attempts to improve the mechanical department of surgery are worthy of attention; but, after all, the treatment of a fractured leg is so very simple a matter, that it would require some ingenuity to mar it; the whole mystery being to lay the leg straight, and keep the toes from turning inward or outward, which may be admirably effected by means of common wooden splints and two or three pieces of tape.

The second paper, *on the Morbid Formation of Bone in the Muscles*, is of considerable interest.

This phenomenon, as occurring in the muscular structure of the heart and blood-vessels, has been already frequently observed and described: its occurrence in the voluntary muscles, however, has attracted less notice. The attention of our author has been chiefly directed to a disease of this nature observed among the Prussian recruits, consisting in a morbid deposition of bone in the muscles on the fore part of the shoulder, and attributed to the pressure of the musket, which induces a species of chronic inflammation, with gradual induration of the parts, terminating in the conversion of a greater or smaller portion of the muscular substance into a bony mass. We are not aware that any such affection is prevalent among our own troops; but it is to be remembered that the German muskets are very much heavier than ours, and Dr. Richter states, that all the recruits suffer more or less from inflammation and swelling of the shoulder, till they become habituated to the exercise. In the greater number these symptoms gradually disappear, without being followed by any permanent bad consequences: every year, however, affords several examples of the disorganization alluded to, which, in our author's opinion, is favoured by friction with spirituous applications, which the men are in the habit of using with a view of allaying the inflammation, but, in reality, with an opposite effect.

"At the commencement of the disease," says our author, "the bony tumors present themselves in the form of knots under the skin, of the size of a hazel-nut; they feel elastic and cartilaginous, are somewhat movable, and are not circumscribed, but blend imperceptibly with the surrounding inflammatory swelling. These knots afford nuclei for further morbid deposition in the contiguous parts already degenerated in their texture. The tumor increases from day to day, becomes continually harder, and, as the inflammation subsides, more circumscribed; it assumes an oval form, and is produced at its lower end into a hard cord, which is afterwards ossified, and appears like a stem in the tendon of the deltoid. In the course of from four to six weeks I have seen the bony deposition increase in this manner to the size of a goose's egg. The motion of the arm is of course impeded by this degeneration. During the inflammatory stage the patient cannot use the limb with freedom: when, however, this is past, and the hardened tumor has acquired only the magnitude of a walnut, the soldier is not prevented from the exercise of his duty, and complains of little inconvenience, unless he be ill-disposed, and wish to avail himself of his mishap as a ground of exemption from further service. If the tumor has attained a larger size, and extended itself to the coraco-brachialis and pectoralis major upward to the clavicle, the elevation of the arm, and its lateral movements, become seriously impeded."

Tumors of this kind may be extirpated, or their resolution may be attempted by discutient applications: our author's experience is unfavourable to the former procedure. He relates the cases of two soldiers from whom he removed such tumors, under the impression that they were separate formations in the intermuscular cellular membrane. Though the wound occasioned by the operation healed readily by the first intention in both these cases, it was nearly a year before either of the men was able to handle his musket. For a long time, also, changes of the weather occasioned severe pains and feebleness of motion in the limb. An examination of the extirpated tumors sufficiently explained the cause of these evil consequences, by demonstrating that the parts removed consisted of the altered structure of the muscles themselves.

"By the extirpation of the tumor in both the above-mentioned patients," says Dr. Richter, "I was enabled to investigate the nature of the excised mass, and to convince myself of my error in supposing it to be a parasitical growth, or osseous deposition in the cellular membrane between the muscles. The operation itself removed my first impression, and an examination of the textures of the tumors led to a different conclusion. External examination had induced me to believe them of smaller dimensions than I afterwards found them to be; for they reached even to the humerus, without however adhering to it, and their chief extension was in the direction of their depth. After extirpation, their magnitude was found to equal that of a moderate-sized hen's egg, and their greatest circumference at their middle was from four to five inches. At both ends, but especially the lower, they were produced to a point, and their long diameter was from four to five inches. The surface was uneven, and here and there beset with hooks. The muscular fibres adhered closely to the whole, entered into the solid substance of the mass, and could be separated only with difficulty. At particular parts the surface was invested with a tendinous membrane, which seemed to be traversed by muscular fibres. When the surface was cleansed from all adherent substances, it appeared rough, and not like that of a bone. After the extirpated tumors had lain some time in spirit, they appeared smaller, and somewhat shrunk. The weight was not proportionate to the bulk, for the smaller mass weighed, after this time, ʒij. ℈i., and the larger ʒv. gr. xxx. The consistence of the two was not similar; the larger was harder, and required a saw for its division; the smaller might be divided, though with some effort, by means of a sharp knife. An examination of their substance shewed that it was not homogeneous; the foundation was of muscular substance, which exhibited itself very manifestly in their texture and colour, and in particular parts had a silvery and tendinous, or rather fibrous appearance. In the midst of these longitudinal muscular and tendinous fibres, I found a hard, bony, and porous substance much resembling pumice-stone, but harder, and which might be compared to the dense texture in the ends of long bones. No cellular structure, nor the presence of any substance resembling the marrow of the bones, could be detected in the texture of this new product."

"The hook-shaped bodies on the surface were connected with the bony mass by a tendinous substance, and were movable."

Experience being unfavourable to the extirpation of such tumors, our author recommends a discutient plan of treatment.

If the swelling of the shoulder be still inflammatory, the disease recent, and the induration small, leeches are to be applied, followed by cooling lotions. When the redness has disappeared, mercurial ointment is to be rubbed in, and a mercurial plaster applied during the night. If the disease be further advanced, the inflammatory stage already passed, and the tumor free from pain, friction with mercurial ointment, and stimulating liniments, may be had recourse to from the beginning. The Ung. Hydriod. Potass. is also applicable to this stage of the disease; in which benefit is also to be derived from the continued irritation of blisters and the tartar-emetic ointment.

The success of this treatment is various: applied in the early stage, it will generally effect a complete cure. If the disease be of long standing, and the tumor have attained the size of a walnut, it may generally be diminished, though not entirely got rid of. In some cases, when the tumor is diminished to a certain point, the patient recovers the free use of his arm: in others, where the tumor is so large as to reach nearly to the coracoid process, this fortunate result is not to be hoped for, and the man becomes incapacitated for further service.

The first two papers in this volume are the most worthy of attention, as containing information in some measure new: our notice of the rest must be more cursory.

In the essay *on the Sanative Process of Nature in Introsusception*, the author gives an excellent view of the manner in which the invaginated portion of intestine is separated and cast off. A case is also introduced, in which, when the patient appeared to be at death's door, a sudden amelioration of the symptoms took place on the expulsion of a portion of the small intestine, forty inches in length; and a perfect recovery ensued.

The subject of the next paper, *Chronic Rheumatism of the Elbow-Joint*, obtruded itself most disagreeably on our author's attention, by taking up its residence in his own elbow for thirteen years. Dr. Richter enters fully and judiciously into the treatment of this affection; but, when all is said that can be said, nobody knows what chronic rheumatism is; and, what is worse, we fear nobody knows how to treat it.

Our author's remarks on the *Interstitial Absorption of Bones* are well worthy of perusal. We can here give little more than the heads of the subject.

1. Of interstitial absorption as a general disease, arising from some constitutional malady.

This may be called *tabes ossium*, an affection on which surgical writers have hitherto bestowed little attention. It is characterized by diminution of the volume, or attenuation of the substance of the bone, and occasionally by both. It occurs superficially, or in the inner texture; and, especially when local, sometimes occasions a solution of continuity. It may present itself throughout the whole osseous system, in the bones of a single limb, or in individual bones, or portions of bones. In cases where one half of the body is crippled, the bones of that half are sometimes thus affected.

The disease is most usually confined to a single bone, and the destruction of substance may proceed from within outwards, or *vice versâ*. In pathological museums we find bones which surprise us by their extraordinary porosity, thinness, and levity. Our author has seen calvariæ so thin as to be transparent, and long bones which had scarcely any weight, and consisted only of an external lamina no thicker than paper: the outer surface was smooth, of a shining white, and not porous; the inner surface was rough, and the cancellated structure so completely obliterated, that the bone resembled a hollow tube. The prominences to which the muscles had been attached were scarcely perceptible. In the broad bones, the cancellated structure seemed to be more subject to absorption than the outer shell, and the destructive process was frequently considerably advanced before it became evident externally. This absorption taking place in the parenchyma of the bones, and thereby increasing their porosity, has been called rarefaction of the bones, osteo-porosis, or osteo-spathyrosis, of which fragility of the bones is a symptom. This consumption of the bones rarely occurs as an idiopathic affection, and, where it is the consequence of any other constitutional disease, it is, of course, accompanied with the peculiar symptoms of that disease.

The general causes of this malady enumerated by our author are—

1. Old age.
2. The great class of tabid diseases.
3. Crippling of a limb or one half of the body.
4. Syphilis, and mercurial cachexy.
5. Scrofula.
6. Gout.
7. Rheumatism.
8. Scurvy, cancer, and herpetic cachexy.

II. Of interstitial absorption as a local disease arising from pressure.

The causes which operate in its production are—

1. Aneurisms.
2. Fungous tumours of the brain, and its membranes.
3. Tumours in the nose and antrum maxillare.
4. Hydrocephalus.
5. The attrition of the act of chewing with a jaw that has lost its teeth.

6. The action of the muscles in various curvatures of the spine.

7. Contusion of the bones.

8. Some other mechanical causes.

III. Of interstitial absorption as an exertion of the sanative power of nature.

This is exemplified—

1. In the healing of fractures, where inequalities in the bone are removed by the action of the absorbents.

2. In the restoration of the medullary structure in the seat of the fracture, the cavity for containing the marrow being, for several months after the reunion of the fracture, obliterated by bony matter, which is afterwards removed by the absorbents, and the natural structure restored.

3. In the diminution of the quantity of callus.

4. In the removal of portions of bones by the saw, as in amputation; the sharp edges of the truncated bone being removed by the absorbents, the cavity filled up by new deposition, and the end of the bone brought into a rounded form.

5. In necrosis.

6. In the formation of new articular cavities in cases of unreduced dislocation.

7. In the changing of the teeth.

Of the cases which constitute the sixth paper, two afford examples of inflammation of the diaphragm; in the one instance rheumatic, in the other, consecutive on softening of the brain: these we would gladly extract, but that they are too diffuse.

The two concluding papers are excellent of their kind, affording an useful compendium of the observations and opinions of authors on two interesting subjects: since, however, they contain comparatively little original matter, we shall not protract this article by any quotations from them.

In conclusion, we beg to recommend all these tracts to the attention of those of our readers who are conversant with German: they are the production of a well-informed and judicious physician.

